# Differences in the coronal plane alignment of the knee (CPAK) measurements using long-leg radiographs and computed tomography

**DOI:** 10.1007/s00402-026-06463-5

**Published:** 2026-08-11

**Authors:** Kohei Kawaguchi, Mei Lin Tay, Simon Young

**Affiliations:** 1 Department of Surgery, University of Auckland, Auckland, New Zealand; 2 Department of Orthopaedic Surgery, The University of Tokyo, Tokyo, Japan; 3 Department of Orthopaedic Surgery, North Shore Hospital, Auckland, New Zealand

**Keywords:** Coronal alignment, Long-leg radiograph, Computed tomography, Coronal plane alignment of the knee (CPAK), Total knee arthroplasty

## Abstract

**Introduction:**

Coronal plane alignment of the knee (CPAK) parameters are increasingly used to characterize native coronal alignment and guide alignment strategies in total knee arthroplasty (TKA) based on long-leg radiographs (LLR). Computed tomography (CT) based robotic TKA software enables calculation of CPAK parameters based on anatomical reference points. However, potential differences in CPAK measurements between LLR and CT remain incompletely defined. This study compared CPAK coronal alignment parameters and phenotype distributions between LLR and CT, and evaluated factors associated with measurement discrepancies.

**Methods:**

This was a radiographic subanalysis of a prospective randomised controlled trial including 241 patients undergoing robotic-assisted TKA. Medial proximal tibial angle (MPTA) and lateral distal femoral angle (LDFA) were measured and arithmetic hip-knee-ankle-angle (aHKA), joint line obliquity (JLO) and CPAK phenotype distribution were calculated and classified from preoperative LLR and CT. Discrepancy groups were defined as LLR = CT (≤ 2°), LLR < CT (> 2°) and LLR > CT (> 2°). Associations between discrepancy groups and preoperative deformity and flexion contracture were assessed.

**Results:**

Compared with LLR, CT demonstrated a more varus MPTA (mean 86.7° vs. 87.1°, *p* = 0.04), a more valgus LDFA (87.1° vs. 87.8°, *p* < 0.01) and a lower JLO (173.9° vs. 174.9°, *p* < 0.01). The aHKA did not differ between modalities. There was a higher overall proportion of apex distal phenotypes (Types I–III) on CT (*p* = 0.01), and in particular CPAK Type II phenotypes were more frequent on CT than LLR (45.2% vs. 33.2%; *p* < 0.01). For MPTA, the proportions of LLR = CT, LLR > CT, and LLR < CT were 66.0%, 21.6% and 12.4%, respectively; corresponding LDFA proportions were 84.6%, 13.3% and 2.1%. In MPTA discrepancy, LLR > CT groups showed more preoperative valgus alignment and LLR < CT group showed more preoperative varus alignment with knee flexion contracture.

**Conclusion:**

CPAK coronal alignment parameters and phenotype distributions differ depending on whether measurements are derived from LLR or CT. Discrepancies in MPTA are influenced by preoperative coronal knee alignment and flexion contracture. Surgeons should interpret CT- and LLR-based CPAK assessments with caution, particularly in patients with moderate deformity or flexion contracture.

**Level III:**

Retrospective Cohort study.

## Introduction

Native coronal knee alignment varies widely between individuals, and several studies have suggested that restoring a patient’s pre-arthritic alignment, rather than enforcing neutral mechanical alignment, may improve postoperative outcomes following total knee arthroplasty (TKA) [1–4]. The Coronal Plane Alignment of the Knee (CPAK) classification system was introduced to characterise coronal knee alignment using two parameters: the arithmetic hip-knee-ankle angle (aHKA) and joint line obliquity (JLO) [5]. Based on these parameters, knees are categorised into nine phenotypes, providing a simple and clinically useful framework for describing native coronal alignment. Importantly, restoration of the preoperative CPAK phenotype has been associated with improved patient-reported outcomes following TKA [6, 7].

**Fig. 1:**
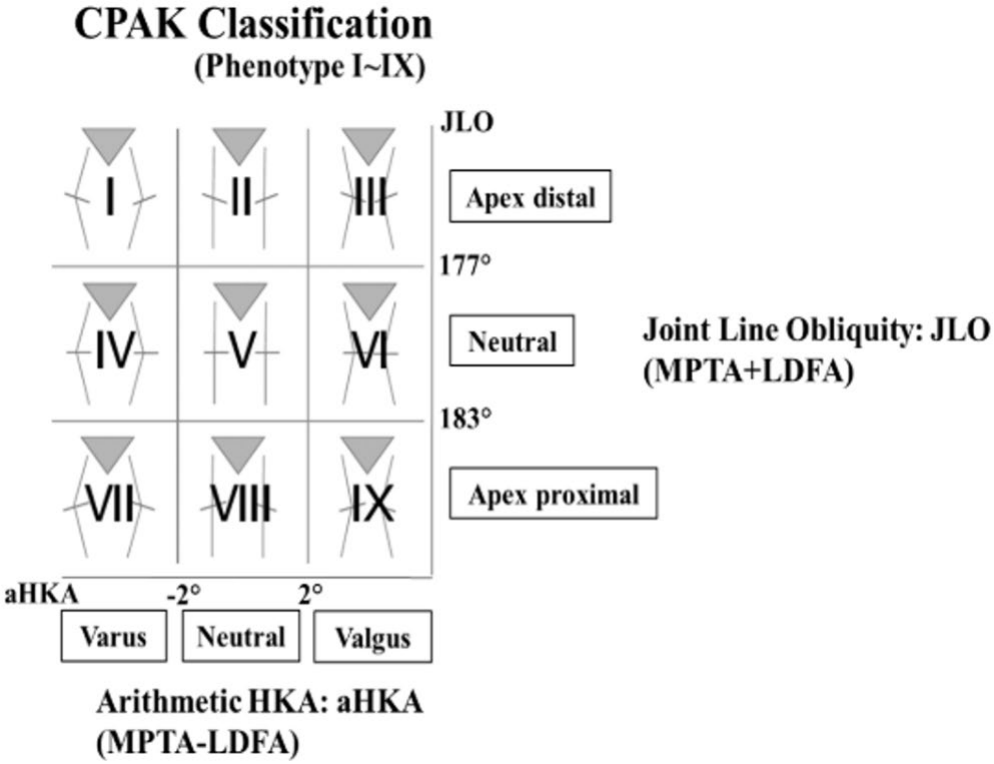
Diagram of the Coronal Plane Alignment of the Knee (CPAK) classification system from MacDessi et al. [20] showing nine knee phenotypes defined by three joint line obliquity (JLO) categories and three arithmetic hip–knee–ankle angle (aHKA) categories. MPTA, medial proximal tibial angle; LDFA, lateral distal femoral angle

The CPAK classification was originally developed using standing, double-limb long-leg radiographs (LLR), with the medial proximal tibial angle (MPTA) and lateral distal femoral angle (LDFA) measured directly, and aHKA and JLO calculated from these values [5] (Fig. 1). However, with the increasing adoption of robotic-assisted TKA, particularly image-based systems, preoperative coronal alignment assessment is now frequently performed using computed tomography (CT) [8, 9]. CT-based imaging provides detailed three-dimensional assessment of bony morphology and forms the basis for preoperative planning and intraoperative execution during robotic-assisted TKA [10–12]. As a result, CPAK measurements derived from CT are increasingly used in clinical practice, despite the classification having been originally validated using LLR [8].

Previous studies comparing CPAK parameters measured on LLR and CT have produced inconsistent findings [13–15]. Some authors have reported that LLR underestimates varus alignment and joint line obliquity compared with CT, while others have reported the opposite or have not performed direct statistical comparisons [13–15]. Moreover, prior CT-based studies have used heterogeneous measurement techniques that were not based on robotic planning algorithms, and the underlying reasons for discrepancies between LLR and CT measurements remain poorly understood [13, 14]. In particular, the influence of preoperative coronal deformity and knee flexion contracture on these discrepancies has not been clearly defined.

Therefore, the aims of this study were to (1) compare CPAK coronal alignment parameters and phenotype distributions measured using LLR and CT in patients undergoing TKA, and (2) identify factors associated with discrepancies between LLR- and CT-based measurements of MPTA and LDFA, with particular emphasis on preoperative coronal knee deformity and knee flexion contracture.

## Patients and methods

This study was a retrospective sub-analysis of data derived from a prospective randomized controlled trial [16, 17]. A total of 236 patients (241 knees) who underwent robotic-arm assisted TKA using a cruciate-retaining Triathlon knee system (Stryker, Fort Lauderdale, FL, USA) at a single institution between November 2020 and December 2022 were included.

**Fig. 2:**
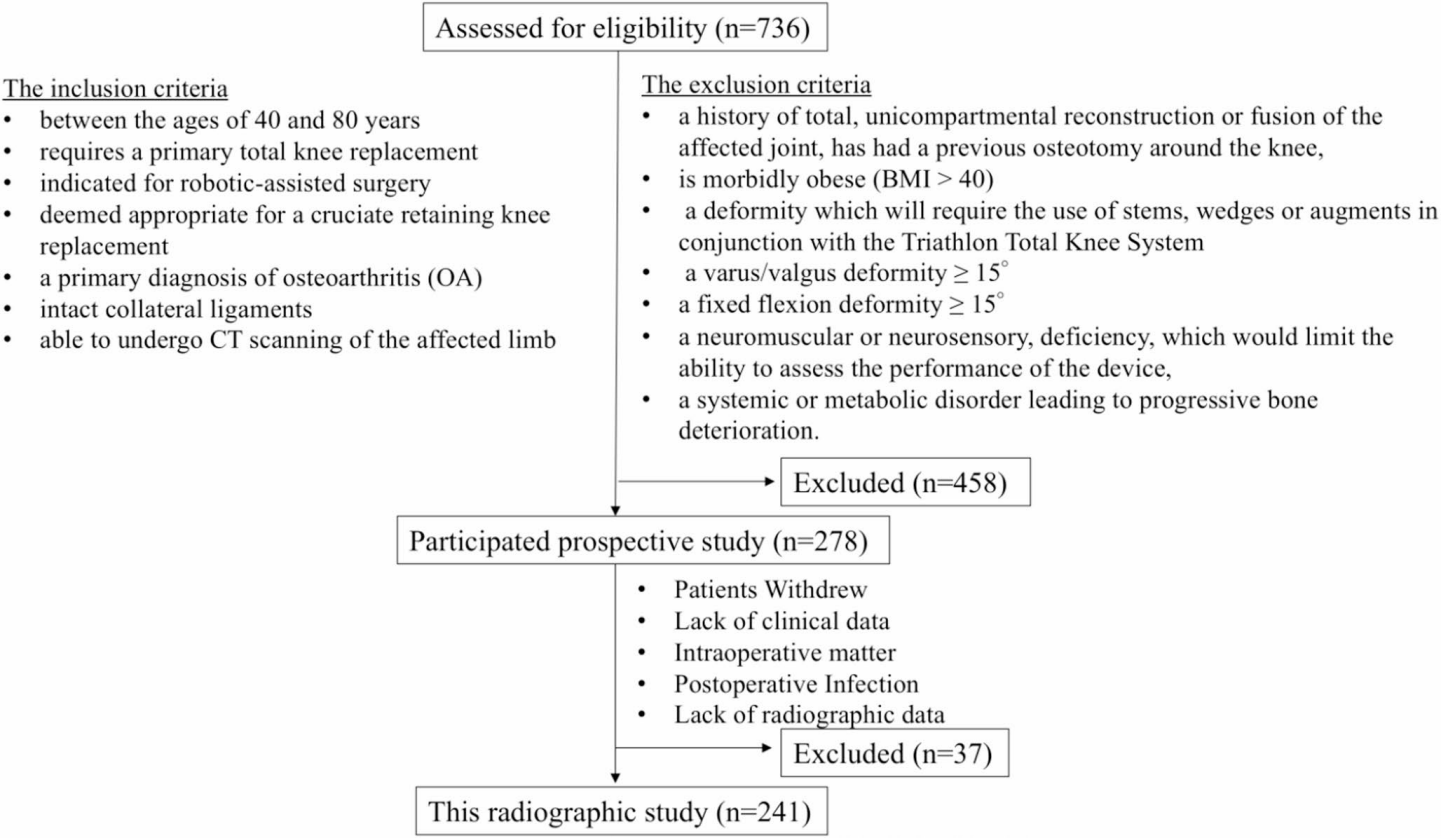
Study flow chart. BMI: body mass index

Patients were eligible for inclusion in the original trial if they were aged between 40 and 80 years with a primary diagnosis of osteoarthritis. Exclusion criteria included previous total or partial reconstruction, fusion, or osteotomy of the affected knee; a body mass index (BMI) > 40 kg/m²; deformities requiring the use of stems, wedges, or augments; varus/valgus deformity ≥ 15°; fixed flexion deformity ≥ 15°; or immunosuppression. For the purposes of this subanalysis, patients were included if they had both preoperative long-leg radiographs (LLR) available and no postoperative complications affecting radiographic assessment (Fig. 2). Preoperative range of knee motion was evaluated using a goniometer by surgeons. Ethical approval was obtained from a national ethical review board (20/NTB/10).

### Radiographic study protocol

Radiographic measurement of coronal alignment parameters and classification into the CPAK phenotypes were performed according to the methodology described by MacDessi et al. [5]. Briefly, MPTA and LDFA were measured on both LLR and CT. The JLO and aHKA were subsequently calculated as JLO = MPTA + LDFA, and aHKA = MPTA – LDFA.

Based on the calculated aHKA and JLO values, knees were classified into CPAK phenotypes I to IX using established cutoff values of ± 2º for aHKA and ± 3º for JLO (Fig. 1). To further investigate discrepancies between imaging modalities, patients were classified into three groups based on the absolute difference between LLR and CT measurements of MPTA or LDFA: LLR = CT (difference ≤ 2°); LLR > CT (LLR value exceeding CT by > 2°); and LLR < CT (CT value exceeding LLR by > 2°). In addition, discrepancies in MPTA and LDFA were analysed according to preoperative knee alignment, classified as neutral, varus, or valgus.

### Plain long-leg radiograph (LLR) measurement

**Fig. 3:**
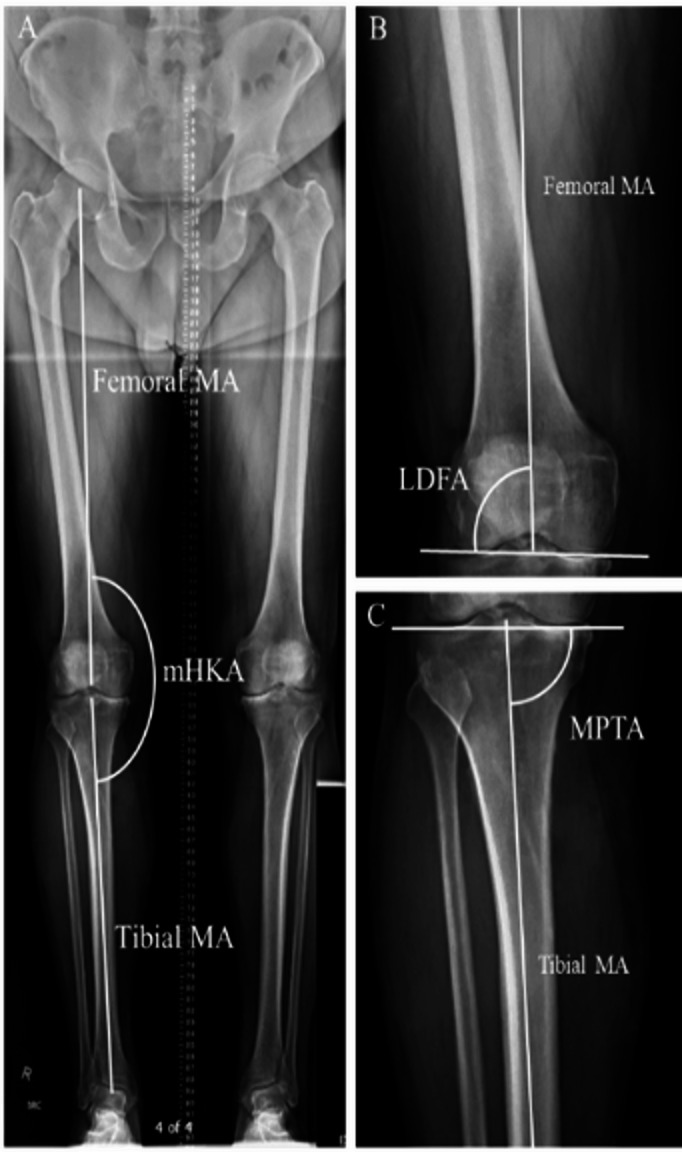
Coronal alignment parameters measured on long-leg radiographs. **A** Mechanical hip–knee–ankle angle (mHKA), defined as the angle between the femoral and tibial mechanical axes. **B** Lateral distal femoral angle (LDFA), defined as the angle between the femoral mechanical axis and the distal femoral joint line. **C** Medial proximal tibial angle (MPTA), defined as the angle between the tibial mechanical axis and the proximal tibial joint line. MA, mechanical axis

Long-leg radiographs were obtained as standing, double-limb radiographs with both patellae facing forward. Measurements were performed according to the CPAK methodology described by MacDessi et al. [5]. The mechanical hip-knee-ankle (mHKA) angle was defined as the angle between the mechanical axes of the femur and tibia. LDFA was defined as the lateral angle between the femoral mechanical axis and the distal femoral joint line, while MPTA was defined as the medial angle between the tibial mechanical axis and the proximal tibial joint line (Fig. 3).

All radiographs were processed digitally and measured using XERO universal software (AGFA Healthcare, Mortsel, Belgium).

### Computed tomography measurement

Preoperative CT scans were performed according to the Mako Total Knee application protocol (Mako TKA 1.0 software; Stryker). For CT measurements, coronal alignment parameters were defined to be analogous to those used for LLR and were measured using the MAKO robotic software. Hip, knee and ankle centres were identified based on three-dimensional analysis of CT data.

**Fig. 4:**
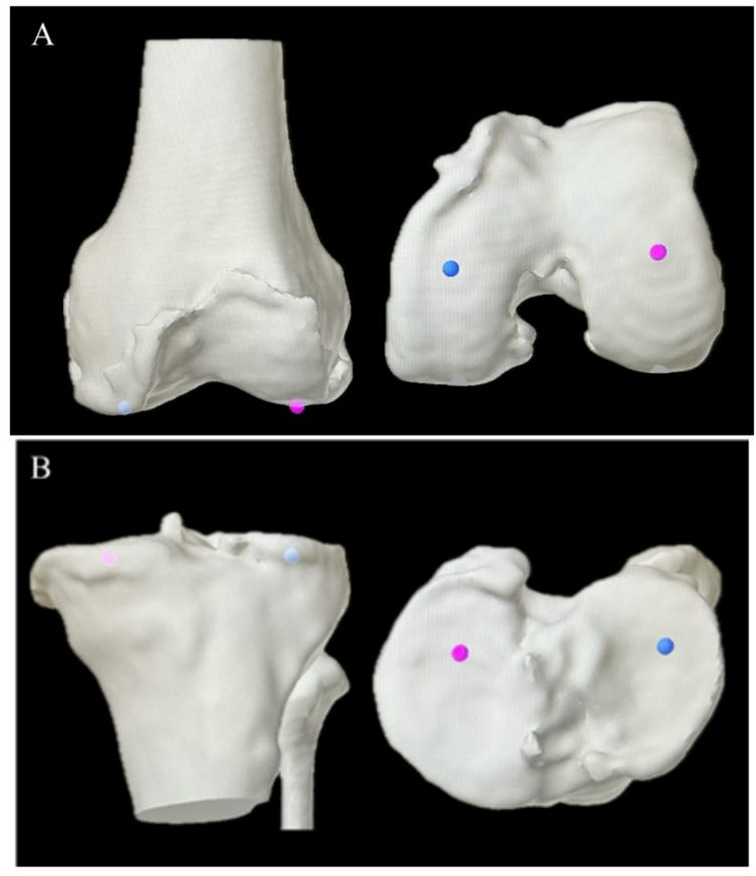
Coronal alignment landmarks on three-dimensional computed tomography reconstructions within the Mako Total Knee application protocol (Mako TKA 1.0 software; Stryker). **A** Distal femoral landmarks defined at the most distal surface of the medial and lateral femoral condyles. **B** Proximal tibial landmarks defined at the 66% point, located two-thirds of the distance from the anterior to the posterior cortex on the medial and lateral tibial plateaus

Femoral reference points were defined at the most distal surface of the medial and lateral femoral condyles. Tibial reference points were defined at the “66% point”, located two-thirds of the distance from the anterior to the posterior cortex on both the medial and lateral tibial plateaus. This point represents the region of maximal contact stress and approximates the dwell point of the tibial insert [15] (Fig. 4). LDFA was defined as the lateral angle between the femoral mechanical axis and the line connecting the distal femoral reference points. The femoral coronal plane was defined using the medial and lateral distal femoral reference points and the center of the femoral head. MPTA was defined as the medial angle between the tibial mechanical axis and the line connecting the proximal tibial reference points. The tibial coronal plane was defined using the medial and lateral proximal tibial reference points and the ankle center.

As with LLR measurements, aHKA and JLO were calculated from MPTA and LDFA values, and knees were classified into CPAK phenotypes I to IX according to established criteria [5].

### Statistical analysis

Statistical analyses were performed using SPSS version 26 (IBM Corp, Armonk, NY, USA). The normality of data distribution was determine using the Shapiro–Wilk test. Normally distributed data were presented using mean (± standard deviation). Paired t-tests were used to compare MPTA, LDFA, aHKA, and JLO measurements between LLR and CT. Correlations between LLR- and CT-derived measurements were assessed using Pearson’s correlation coefficient. An agreement between two measurements were evaluated by Lin’s concordance correlation coefficient. Categorical variables were compared using chi-square tests. Bland-Altman plots were utilized to assess the agreement between LLR and CT methods, to visualize outliers, and to detect potential systematic bias. These plots illustrate the 95% limits of agreement, determined by the mean difference ± 1.96 standard deviations and indicate the expected range of agreement within which the measurements from both methods were anticipated to fall.

Repeated measures analysis of variance (ANOVA) was used to compare coronal alignment parameters among the three discrepancy groups, with Bonferroni correction applied for multiple comparisons. Statistical significance was set at *p* < 0.05. Post hoc power analysis demonstrated statistical power (1 − β) of 0.86 for MPTA and 1.0 for LDFA. Inter- and intra-observer reliability for LLR measurements of MPTA and LDFA were assessed in a randomly selected subgroup of 20 radiographs using intraclass correlation coefficients (ICC). Intra-observer measurements were repeated at a three-week interval.

## Results

Demographic characteristics and preoperative radiographic data of the 241 patients included in this study are presented in Table 1. Inter-observer agreement between two independent orthopaedic surgeons was excellent for MPTA (ICC 0.947) and good for LDFA (ICC 0.779). Intra-observer agreement for measurements performed at three-week intervals was excellent for both MPTA (ICC 0.983) and LDFA (ICC 0.933).

**Table 1:**
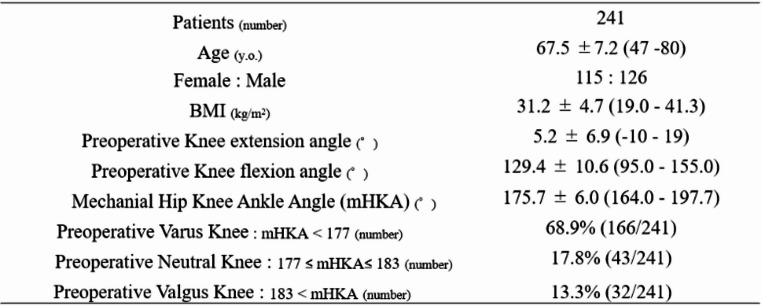
Demographic and preoperative radiographic parameters

Data are presented mean ± standard deviation (range)

BMI, body mass index; mHKA, mechanical hip knee ankle angle

**Fig. 5:**
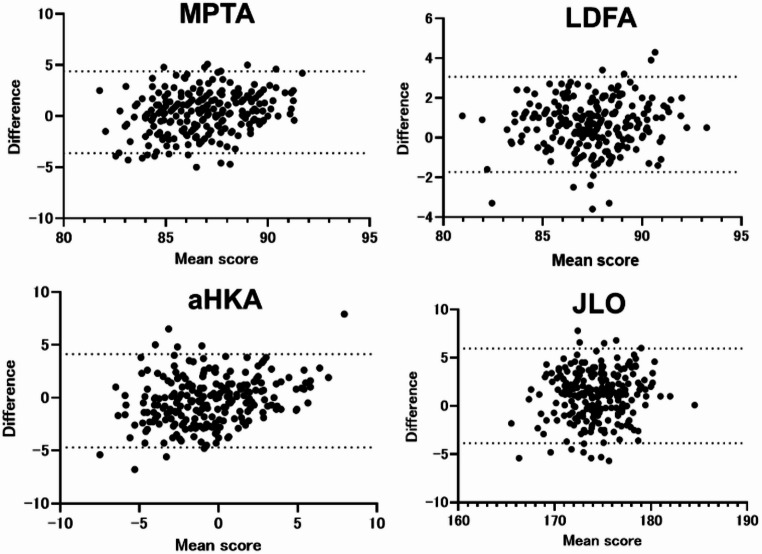
Bland-Altman plots for the MPTA, LDFA, aHKA, and JLO. The dot black lines represent the 95% limits of agreement (± 1.96 Standard deviations). MPTA, medial proximal tibial angle; LDFA, lateral distal femoral angle; JLO, joint line obliquity; aHKA, arithmetic hip–knee–ankle angle

Compared with LLR, the CT measurements demonstrated a more varus MPTA (86.7°± 2.1 vs. 87.1° ±2.5, *p* = 0.04; Table 2), and a more valgus LDFA (87.2° ± 2.1 vs. 87.8° ± 2.2, *p* < 0.01). The JLO measured using CT showed greater distal apex obliquity to the joint line compared with LLR (173.9° ± 3.1 vs. 174.9^°^ ± 3.4, *p* < 0.01). There was no significant difference in aHKA between imaging modalities. There was a strong positive correlation between LLR and CT for LDFA (correlation coefficient (CC) 0.836, *p* < 0.01; Table 2) and a moderate positive correlation for MPTA, aHKA, and JLO (CC 0.626, 0.741, 0.706, respectively; all *p* < 0.01). Figure 5 illustrates the Bland-Altman plots for the different variables. The percentage of observations that fell within the limits of agreement was 93.8% for the MPTA, 95.9% for the LDFA, 96.2% for the aHKA, and 94.6% for the JLO.

**Table 2:**
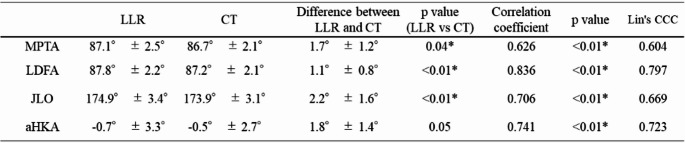
Comparison of coronal alignment parameters measured on long-leg radiographs and computed tomography

Data is presented average ± standard deviation. MPTA, medial proximal tibial angle; LDFA, lateral distal femoral angle; aHKA, arithmetic hip-knee-ankle angle; JLO, joint line obliquity; LLR, long-leg radiograph; CT, computed tomography; Lin’s CCC, Lin’s Concordance Correlation Coefficient

*: significant difference (*p* < 0.05)

**Fig. 6:**
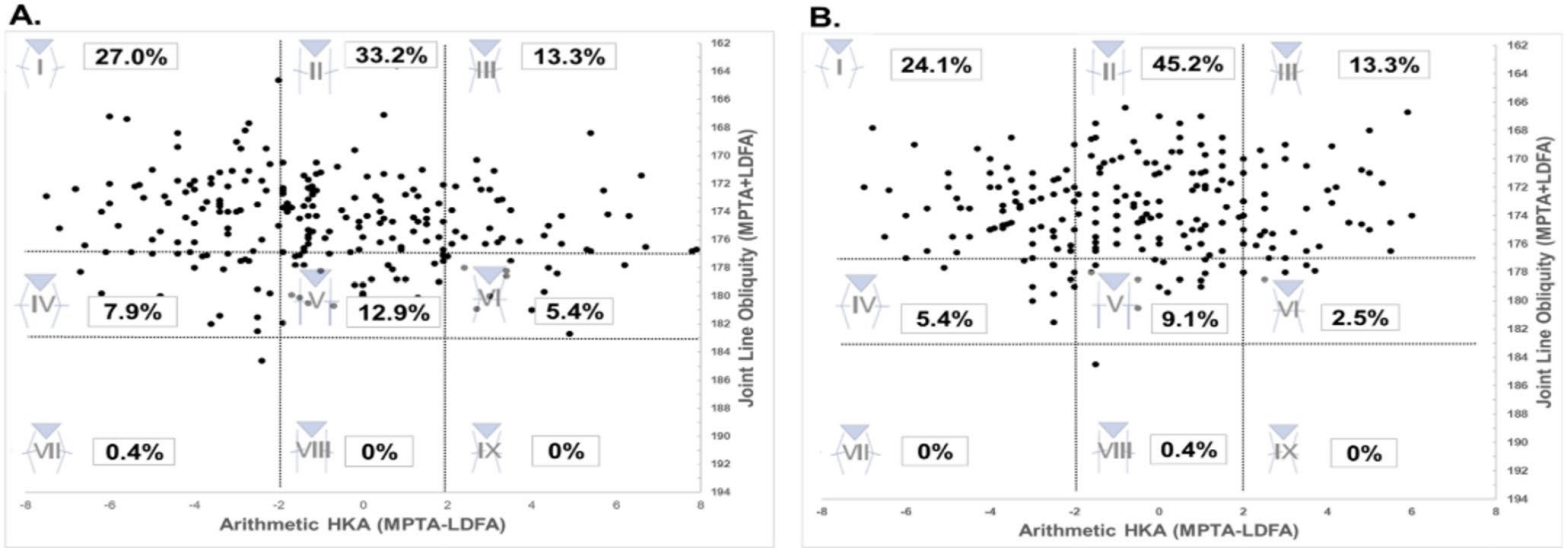
Distribution of Coronal Plane Alignment of the Knee (CPAK) phenotypes. **A** Scatter plot of arithmetic hip–knee–ankle angle (aHKA) versus joint line obliquity (JLO) measured on long-leg radiographs. **B** Corresponding distribution measured on computed tomography. Percentages represent the proportion of knees in each CPAK phenotype. MPTA, medial proximal tibial angle; LDFA, lateral distal femoral angle; JLO, joint line obliquity; aHKA, arithmetic hip–knee–ankle angle

Analysis of CPAK phenotype distribution demonstrated a higher proportion of Type II phenotypes when measured using CT compared with LLR (45.2% vs. 33.2%; *p* < 0.01; Fig. 6; Table 3). Overall, CT-based measurements demonstrated a higher proportion of apex distal JLO phenotypes (CPAK Types I, II, and III) compared with LLR (*p* = 0.01).

**Table 3:**
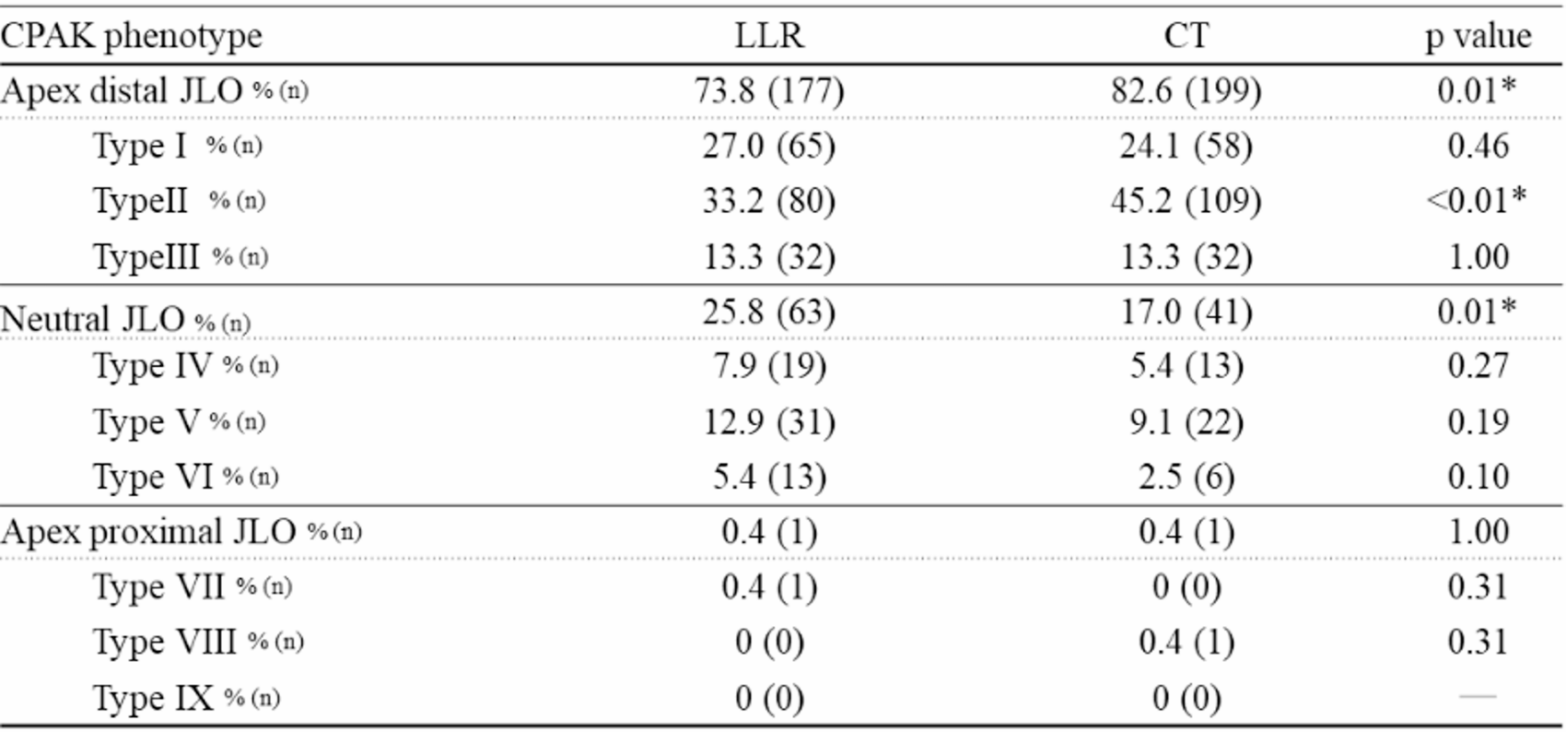
Distribution of Coronal Plane Alignment of the Knee (CPAK) phenotypes measured on long-leg radiographs and computed tomography

CPAK, Coronal Plane Alignment of the Knee; LLR, long-leg radiograph; CT, computed tomography; JLO, joint line obliquity

*: significant difference (*p* < 0.05)

**Fig. 7:**
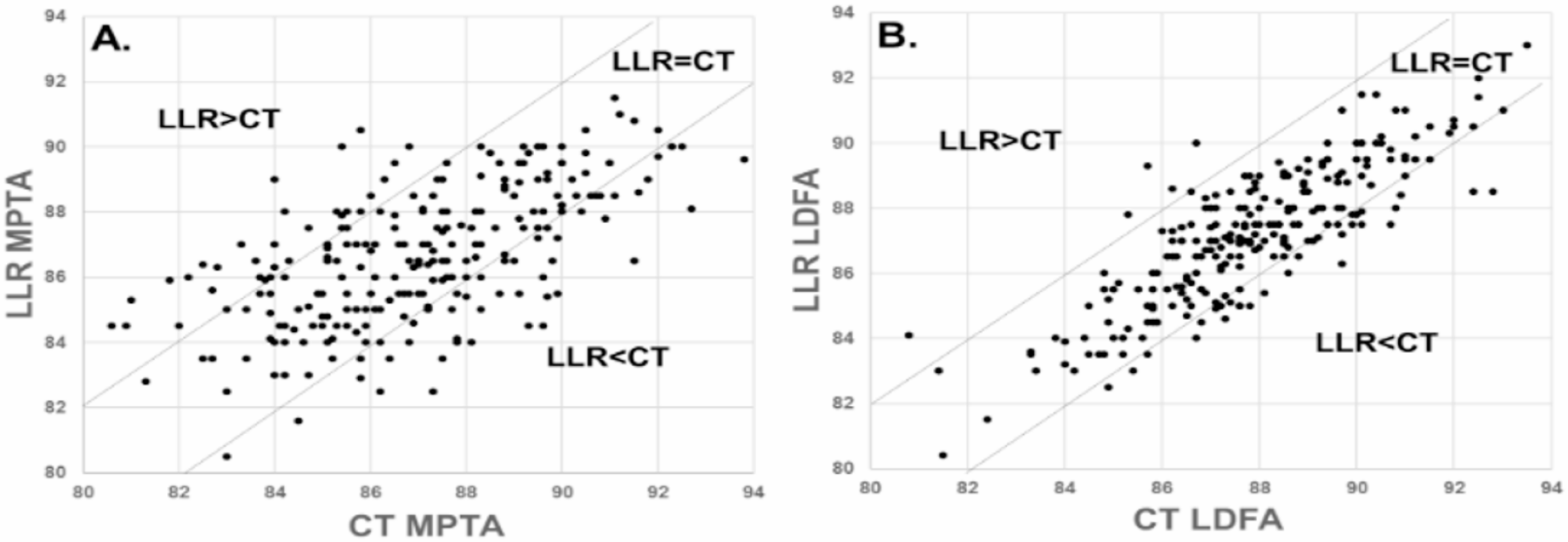
Comparison of coronal alignment measurements between long-leg radiographs (LLR) and computed tomography (CT). **A** Medial proximal tibial angle (MPTA). **B** Lateral distal femoral angle (LDFA). Labelled regions indicate agreement within 2° (LLR = CT), LLR values > 2° greater than CT (LLR > CT), and CT values > 2° greater than LLR (LLR < CT). MPTA, medial proximal tibial angle; LDFA, lateral distal femoral angle; JLO, joint line obliquity; aHKA, arithmetic hip–knee–ankle angle

Patients were further categorized into three groups based on the absolute difference between LLR and CT measurements of MPTA and LDFA (Fig. 7). For MPTA, the LLR > CT group demonstrated a larger mHKA compared with the other two groups (*p* < 0.01) while the LLR < CT group demonstrated greater preoperative flexion contracture and lower mHKA compared with the other two groups (*p* = 0.03, and *p* < 0.01 respectively; Table 4). In contrast, for LDFA, the only differences observed between groups were the LDFA values themselves, with no differences in other alignment parameters or demographic variables (Table 5).

**Table 4:**
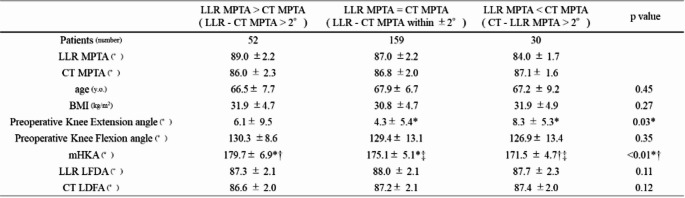
Comparison of patient characteristics and alignment parameters according to discrepancies between long-leg radiograph and computed tomography measurements of medial proximal tibial angle (MPTA)

LLR, long-leg radiograph; CT, computed tomography; mHKA, mechanical hip knee ankle angle; LDFA, lateral distal femoral angle; MPTA, medial proximal tibial angle; BMI, body mass index

*†‡: significant difference (*p* < 0.05)

**Table 5:**
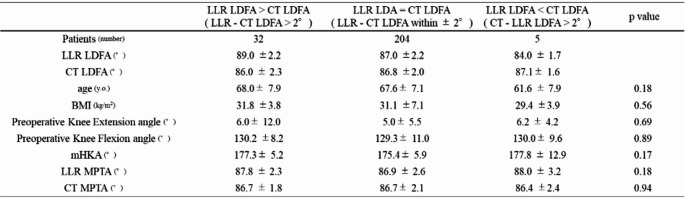
Comparison of patient characteristics and alignment parameters according to discrepancies between long-leg radiograph and computed tomography measurements of lateral distal femoral angle (LDFA)

LLR, long-leg radiograph; CT, computed tomography; mHKA, mechanical hip knee ankle angle; MA, mechanical axis; LDFA, lateral distal femoral angle; MPTA, medial proximal tibial angle; BMI, body mass index

*†‡: significant difference (*p* < 0.05)

**Fig. 8:**
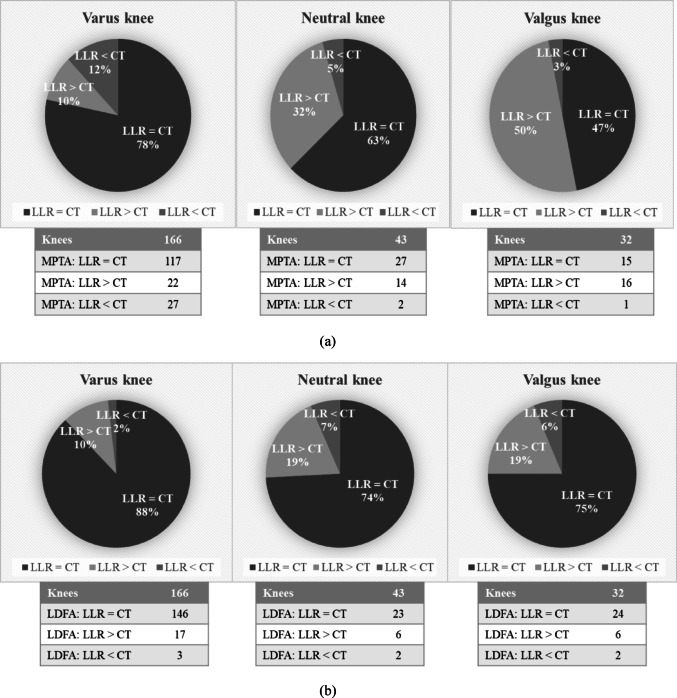
Association between preoperative coronal knee alignment and discrepancies between long-leg radiograph (LLR) and computed tomography (CT) measurements of medial proximal tibial angle (MPTA) and lateral distal femoral angle (LDFA). Varus knees were defined as preoperative mHKA > 3° varus, neutral knees as − 3° to + 3°, and valgus knees as > 3° valgus. Discrepancy categories were defined as agreement within 2° (LLR = CT), LLR > CT (> 2°), and LLR < CT (> 2°)

When stratified by preoperative coronal knee alignment, discrepancies in MPTA measurements differed between varus, neutral, and valgus knees (Table 6). For MPTA, LLR < CT discrepancies occurred in 90% of varus knees, while LLR > CT discrepancies were observed in all alignment groups. Approximately 50% of the valgus knees demonstrated an LLR > CT discrepancy in MPTA (Fig. 8a). There were no differences in LDFA among the three groups (Fig. 8b).

**Table 6:**
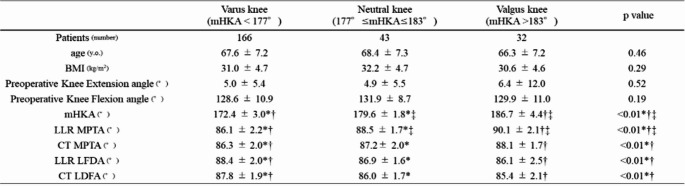
Characteristics of three groups according to preoperative coronal knee deformity

LLR, long-leg radiograph; CT, computed tomography; mHKA, mechanical hip knee ankle angle; MA, mechanical axis, LDFA, lateral distal femoral angle; MPTA, medial proximal tibial angle; BMI, body mass index

*†‡: significant difference (*p* < 0.05)

## Discussion

The principal finding of this study was that CPAK coronal alignment parameters and phenotype distributions differed depending on whether measurements were obtained using long-leg radiographs or computed tomography. In addition, preoperative coronal knee alignment and knee flexion contracture were associated with discrepancies in MPTA measurements between LLR and CT.

We found that MPTA measured using CT was more varus (smaller) compared with measurements obtained from LLR. Only two previous studies have compared CPAK parameters between LLR and CT [14, 15]. Tarassoli et al. reported smaller MPTA when measured using CT compared with LLR, consistent with our findings [15]. In contrast, León-Muñoz et al. reported larger MPTA on CT compared with LLR [14]. These discrepancies between study findings may be attributable to differences in CT measurement methodology. In particular, León-Muñoz et al. used a manually defined line connecting the deepest points on the tibial plateau for the MPTA, whereas the present study utilized the “66% point” detected automatically using a computer algorithm within the MAKO system. Additionally, the present study demonstrated substantial individual variation in MPTA differences between LLR and CT, whereas previous studies focused on aggregate analyses and did not account for individual differences in each case. Specifically, while overall analysis in this study showed that MPTA measured using LLR was larger than MPTA measured with CT, the proportion of knees where LLR > CT MPTA (where the difference exceeded 2°) was only 21.6%. The proportion of knees where LLR < CT MPTA was 12.5%. This highlights the limitations of focusing on aggregate (mean) differences when comparing alignment measurements between modalities.

Further analysis demonstrated that MPTA discrepancies were influenced by preoperative knee alignment and flexion contracture. LLR > CT MPTA discrepancies occurred in both varus and valgus knees, whereas LLR < CT MPTA discrepancies were observed predominantly in varus knees with flexion contractures. For LLR > CT MPTA discrepancies, where varus deformity is underestimated when using LLR, this may be due to differences in the proximal medial tibial reference point in varus knees. The medial reference point in LLR is on the tangent line of the medial sclerotic concave joint, whereas the CT medial reference point is the 66% point, a potentially deeper point in the concave joint medial tibial plateau; potentially leading to a more varus MPTA when measured using CT [15]. In valgus knees, degenerative changes affecting both compartments may alter the apparent joint line on LLR, contributing to similar discrepancies [18]. Conversely, varus knees with flexion contracture may exhibit complex tibial morphological changes and osteophyte formation that influence CT-based reference point detection, potentially resulting in larger MPTA values on CT. These findings suggest that discrepancies between imaging modalities are systematic rather than random, and are influenced by underlying deformity characteristics.

With respect to the femur, we found LDFA measured using LLR was consistently larger than when measured using CT, in keeping with previous reports [14, 15]. Nearly all LDFA discrepancies favoured LLR > CT, with LLR < CT discrepancies being rare (2.1% of cases). This may reflect the sensitivity of LDFA measurements on LLR to subtle knee flexion and rotation, as CT femoral reference points are generally less affected by degenerative change than those of the tibia. In a simulation study, Brunner et al. reported that knee flexion increased LDFA when measured on LLR, supporting this explanation [19]. In terms of JLO, we found measurements were larger on LLR compared with CT, consistent with findings reported by Tarassoli et al. [15]. This is expected given that JLO represents the sum of MPTA and LDFA, both of which were larger in LLR than CT. In contrast, we found no difference in aHKA measured using LLR and CT Since aHKA represents the difference between MPTA and LDFA, aHKA may be less susceptible to differential femoral and tibial deformities and preoperative flexion contractures.

Analysis of CPAK phenotype distribution demonstrated a higher proportion of apex distal phenotypes (CPAK Type I, II, and III) with CT compared to LLR, particularly for CPAK Type II (LLR: 33.2% vs. CT: 45.2%). This is consistent with the findings of Tarassoli et al., and are likely driven primarily by differences in JLO measurements between imaging modalities [15]. Although there is currently no consensus regarding the most clinically meaningful method for CPAK assessment, these differences are important to recognise, particularly as CT-derived CPAK measurements are increasingly used in robotic-assisted TKA planning.

We found that preoperative knee alignment and knee flexion contracture could explain the discrepancies between MPTA measured using LLR or CT. Fontalis et al. suggested that intra-articular deformity and lower limb positioning during the CT scan could lead to these discrepancies [13], however they did not take into account flexion contracture and coronal knee alignment, and CPAK phenotype distribution was not reported. Brunner et al. assessed the impact of knee flexion and axial rotation angles on MPTA and LDFA in a simulation study. They found that rotation or flexion alone has little impact, however, combined effects of rotation and flexion can reach clinically relevant values [19]. Their study findings were in line with our results. One of the strengths of our research is that it was based on clinical data rather than simulation data. Our findings on the differences between LLR and CT could be helpful in CT-based robotic-assisted TKA, because many clinical TKA studies are based on coronal alignment parameters in CPAK classification in LLR [7, 20, 21]. Therefore, we recommend that surgeons evaluate preoperative coronal knee deformity using not only CT but also LLR when a patient has moderate coronal deformity, and potential discrepancies between LLR and CT should also be taken into consideration.

Several limitations should be acknowledged. First, this was a retrospective radiographic analysis conducted at a single centre, which may limit generalisability. Second, this study deliberately utilised the “66% point” as the tibial reference for CT-based MPTA measurement, as this reference is routinely used with this robotic TKA system, and in a biomechanical study Hazratwala et al. reported that the 66% point closely approximated the weight-bearing point in the native knee [22]. Tarassoli et al. compared various tibial MPTA reference points, and reported the 66% point showed had a more varus MPTA compared to other tibial reference points [15], and alternative reference points may have yield different findings to this study. Third, although our inter- and intra-observer variability were sufficiently reliable, we did not evaluate the factors that may influence these variabilities. Fourth, we did not evaluate sagittal and axial alignment parameters such as tibial posterior slope or femorotibial rotational alignment. Fifth, we did not fully account for differences in the effects of CT and LLR under varying load conditions. Sixth limitation of our study is that we analyzed discrepancies between CT and LLR in MPTA and LFDA using only “2°” threshold. Seventh, although we analyzed four primary alignment parameters (MPTA, LDFA, JLO, and aHKA) as independent anatomical variables with distinct clinical relevance, we acknowledge the potential risk of type I error due to multiple comparisons. As a sensitivity analysis, we applied the Holm–Bonferroni correction to these comparisons. After adjustment, only LDFA and JLO remained statistically significant. Therefore, the present findings should be interpreted with caution, and further studies with larger sample sizes are required to confirm these results. Another important limitation is that the coronal plane may be defined differently on LLRs and CT-based robotic planning software. While LLR measurements are based on a two-dimensional projection of the lower limb, CT-based measurements are derived from three-dimensional anatomical landmarks used to construct the femoral and tibial coronal planes. As a result, MPTA, LDFA, and HKA may not have been assessed in identical planes, which could have introduced measurement bias and contributed to the discrepancies observed between LLR and CT assessments. Finally, while LLR and CT were both obtained using a standardized protocol, radiological measurement errors stemming from rotational malpositioning and knee flexion contractures during image acquisition could not be fully accounted for, and knee flexion and rotation angles were not quantified.

## Conclusion

CPAK coronal alignment parameters and phenotype distributions differ depending on whether measurements are obtained using LLR or CT. Discrepancies in MPTA measurements are influenced by preoperative coronal knee alignment and knee flexion contracture. Surgeons should be aware of these differences and interpret CT- and LLR-based alignment measurements with caution, particularly in patients with moderate coronal deformity or flexion contracture.

## Data Availability

The data that support the findings of this study are not publicly available due to privacy or ethical restrictions.
